# The relationship between gait speed and mediolateral stability depends on a person's preferred speed

**DOI:** 10.1038/s41598-023-32948-z

**Published:** 2023-04-13

**Authors:** Sarah A. Brinkerhoff, William M. Murrah, Jaimie A. Roper

**Affiliations:** 1 School of Kinesiology, Auburn University, Auburn, AL USA; 2 Department of Educational Foundations, Leadership, and Technology, Auburn University, Auburn, AL USA

**Keywords:** Musculoskeletal system, Anatomy

## Abstract

Mediolateral stability during walking requires active control and is complex. Step width, a proxy for stability, follows a curvilinear relationship as gait speeds increase. However, despite the complexity of maintenance for stability, no study has yet investigated the variation across individuals of the relationship between speed and step width. The purpose of this study was to determine if variation between adults affects the estimation of the relationship between speed and step width. Participants walked on a pressurized walkway 72 times. Gait speed and step width were measured within each trial. Mixed effects models assessed the relationship between gait speed and step width, and the variability in the relationship across participants. The relationship between speed and step width followed a reverse J-curve on average, but the relationship was moderated by participants’ preferred speed. Step width response as speed increases is not homogenous in adults. This finding suggests that “appropriate” stability moderation (tested across a range of speeds) differs as a function of an individual’s preferred speed. Mediolateral stability is complex, and further research to elucidate individual factors contributing to variation is needed.

## Introduction

Maintaining mediolateral stability is necessary to sustain an upright posture during walking^1–3^. Mediolateral stability during steady-state unperturbed walking can be measured by step width^2,4,5^, which does not linearly relate to walking speed^6^ but instead follows a reverse J-curve; step width is high at relatively slow and fast speeds and is lowest near preferred speeds^2^. Despite the nonlinear, complex relationship between speed and step width, step width continues to be used in rehabilitation settings as a simple marker for gait health. Therefore, it is important to understand how step width varies across people.

The purpose of this study was to determine how variability between adults affects the estimation of the relationship between speed and stability. We hypothesized that between-participant variability would not affect the within-participant relationship between speed and stability in adults.

## Methods

### Participants

Students aged 19–35 from Auburn University were recruited via word-of-mouth and using a college-wide online study recruitment site that offered course extra credit. Data from these participants have been published previously^7^. Participants did not self-report any lower extremity bone fractures, muscle strains, or joint dislocations in the past six months, nor any chronic pain that affected walking such as pain associated with patellar tendonitis or Osgood-Schlatter Disease. All participants provided written informed consent by reading and signing a consent form approved by the Institutional Review Board of Auburn University. All methods were conducted in accordance with the protocol approved by Auburn University’s Institutional Review Board.

### Procedures

As detailed previously, testing occurred on a 5.8-m-long pressurized walkway (GAITRite, CIR Systems Inc., Clifton, NJ) centered within a 12.1-m laboratory space, and participants began and ended each walking trial at least one stride before and after the walkway^7^. The same research assistant provided 24 different prompts used to elicit a range of walking speeds, participants walked 3 times per prompt, and the order of prompts were randomized across participants (for the list of prompts used, see Brinkerhoff et al.^7^). The prompt was repeated prior to each walking trial. Therefore, we collected and analyzed kinematic data during 72 walking trials per participant.

### Data analysis

**Figure 1:**
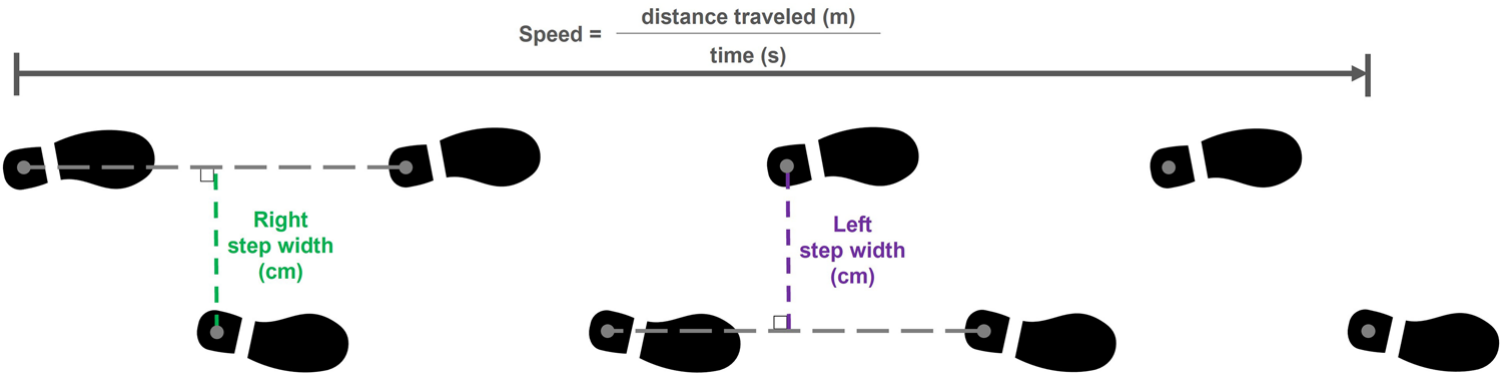
A schematic of how speed and step width were calculated within the GAITRite software. Speed is measured as the distance traveled between the heel center of the first and last footfalls in meters, divided by the time elapsed between first and last footfalls. Step width (termed “HH base of support” in the software) is measured by the distance from the center of the heel to the line of the path of progression between the contralateral steps. A right step width is shown in green, and a left step width is shown in purple.

Speed and step width were exported from GAITRite software. Figure 1 details how speed and step width were calculated within the GAITRite software. Speed was taken as an average per trial. Step width was measured separately for each foot and average per trial (144 step width observations across 72 speeds, per participant). In total, 15 trials were missing from the sample, across 5 participants. Each participant’s preferred speed was determined as the average of their speeds from the prompts, “walk at your normal speed,” and, “walk at your typical speed” were averaged (6 per participant).

Previously, we explored the effect of prompt and individual variance on gait speed using mixed effects models^7^. Here, we explored the effect of gait speed on step width using mixed effects models in R^8–10^. Five separate mixed effects models were built to predict step width and were compared for fit to the data using the AIC, such that a smaller AIC by at least 2 units indicated a better fit to the data^11^. We disaggregated between- and within-participant variation to investigate whether gait speed, as a percent of a participant’s preferred speed, interacted with a participant’s preferred speed to predict step width. To calculate gait speed as a percent of a preferred speed, speed was group-centered within participant on a participant’s preferred speed, and then normalized by calculating trial speeds as percent of preferred speed. Therefore, a participant’s preferred speed was estimated as 0% and speeds above or below their preferred were estimated as a positive or negative percent of preferred speed, respectively. For ease of viewing the table and figure, 100 was added to each speed value so that preferred speed was indicated by 100%.

## Results

Fifty-six adults participated in this study (33 females). Mean age was 21 (SD 3) years and ranged from 19 to 35. Since only two participants were over the age of 25, we ran the same analyses with and without these two participants. The best-fitting model remained the same regardless of inclusion of the older participants. Therefore, we kept all 56 participants in the analysis. Mean leg length was 94 (SD 6) cm, and mean mass was 71.5 (SD 17.5) kg. Mean preferred speed was 1.31 (SD 0.14) m/s and mean speed across all trials was 1.37 (SD 0.32) m/s. Mean step width across all trials was 8.11 (SD 3.93) cm.

The first model included a fixed effect for the mean intercept at percent of preferred speed and a random intercept by participant. The second model was cross-level and built upon the first by adding a fixed interaction between the linear slope of percent of preferred speed and preferred speed; this model tested whether the effect of relative speed on step width was moderated by preferred speed. The second model fit the data better than the first (*X*^2^(3) = 71.644, *p* < 0.001). The third model built upon the second by adding a fixed quadratic effect of speed, to test if the within-participant relationship between speed and step width was curvilinear. The third model fit the data better than the second (*X*^2^(1) = 110.46, *p* < 0.001). The fourth model built upon the third by adding a random slope by participant for the percent of preferred speed. The fourth model fit the data better than the third (*X*^2^(2) = 146.39, *p* < 0.001). The variance in the random intercept and the variance in the random slope were free to correlate. The fifth model added fixed covariates for sex and leg length, to determine if these factors affected step width. The fifth model do not fit the data better than the fourth (*X*^2^(2) = 0.890, *p* = 0.641). The results of each model are provided in Table 1. The intra-class correlation for the fourth and best-fitting model suggested that between-participant differences explained 61% of the variance in step width. The fourth model residuals approximated a normal distribution (upon visual inspection)^12^ based on the QQ plots and plots of the residuals.

**Table 1:** Statistical model results for each of the five models testing the relationship between gait speed and step width.

	Model 1: Intercept-only	Model 2: + linear effect of speed	Model 3: + quadratic effect of speed	Model 4: + random linear slopes	Model 5: + covariates
Intercept	8.10 (0.32) *p* < 0.001	8.29 (3.09) *p* = 0.007	8.15 (3.11) 0.009	8.02 (4.88) *p* = 0.101	11.58 (6.93) *p* = 0.113
Percent preferred speed		−0.09 (0.01) *p* < 0.001	−0.15 (0.01) *p* < 0.001	−0.16 (0.04) *p* < 0.001	−0.16 (0.04) *p* < 0.001
Preferred speed		−0.15 (2.35) *p* = 0.950	−0.15 (2.37) *p* = 0.951	−0.05 (3.72) *p* = 0.990	0.57 (3.94) *p* = 0.888
Percent preferred speed * preferred speed		0.08 (0.01) *p* < 0.001	0.11 (0.01) *p* < 0.001	0.12 (0.03) *p* < 0.001	0.12 (0.03) *p* < 0.001
Preferred speed squared			0.0004 (< 0.001) *p* < 0.001	0.0003 (< 0.001) *p* < 0.001	0.0003 (< 0.001) *p* < 0.001
Sex (female)					−0.60 (0.92) *p* = 0.524
Leg length					−0.04 (0.06) *p* = 0.474
**Random effects**					
Residual variance	9.6363	9.5504	9.4178	9.1120	9.113
Var: ID (intercept)	5.8095	5.7787	5.8749	14.5766	15.70
Var: ID (speed)				0.0008	0.0007873
Corr: intercept*speed				−0.5871	−0.61
ICC	0.3761	0.3770	0.3842	0.6124	0.63
N	56	56	56	56	56
Observations	8021	8021	8021	8021	8021
AIC	41,191.228	41,140.611	41,050.841	40,918.183	40,925.86
log-Likelihood	−20,592.406	−20,556.584	−20,501.353	−20,450.092	−20,428

Values are given in Coefficient (SEM). *p*-values are bolded when *p* < 0.05. The final model is in bold (Model 4).

**Figure 2:**
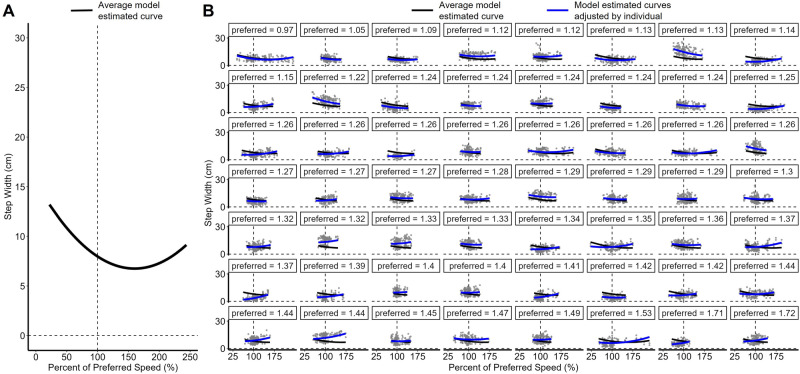
The relationship between percent of preferred speed and step width is a reverse J-curve and is moderated by the magnitude of a participant’s preferred speed. The vertical dashed lines indicate 100% of preferred speed. (**A**) The within-participant relationship between speed and step width, averaged across *n* = 56 participants. (**B**) The relationship between speed and step width for each participant, ordered by increasing preferred speeds to demonstrate the between-participant effect of preferred speed. The black curve is the average model estimated curve, and the blue curves are the intercept- and slope-adjusted model curves (random effects) fit to each individual’s data (gray circles).

According to the fourth model, the estimated mean step width at participants’ preferred speed was 8.02 cm. There was a significant negative linear slope such that as speed increased by 1% of preferred speed, step width decreased by 0.16 cm (*p* < 0.001). There was a significant quadratic slope such that the relationship between speed and step width was convex (*Β* = 0.0003, *p* < 0.001). The negative tilt and convex curve resulted in a reverse J-curve (Fig. 2A). However, the linear slope between percent of preferred speed and step width was moderated by participants’ preferred speed such that those with slower preferred speeds had a larger negative slope between speed and step width, and those with faster preferred speeds had a smaller negative slope (*Β* = 0.12), *p* < 0.001) (Fig. 2B). There was significant random variance in the intercept and in the linear slope across participants, and the random variances were correlated (*r* = −0.59).

## Discussion

The purpose of this study was to determine whether a reverse J-curve fit the relationship between gait speed and step width—as a measure of stability—and whether gait speed interacted with a participant’s overall preferred speed in predicting step width. The negative tilt and convex shape of the relationship between percent of preferred speed and step width substantiates that the within-participant relationship between speed and step width, on average, follows a reverse J-curve^2^ and reaches a minimum around 150% of preferred speed (Fig. 2A). Mediolateral stability is dependent on sensory input, and when instability is recognized, step width tends to increase^13,14^. It may be that at gait speeds higher than 150% of preferred speed, sensory information about mediolateral stability is reduced (or noise is increased), resulting in increased step width in order to ensure swing foot clearance and mediolateral stability^13^. While the within-participant relationship between speed and step with is a reverse J-curve, between-participant variability moderates this relationship.

Figure 2B displays the participant-level data in order of increasing preferred speed and demonstrates the model interaction between preferred speed and the linear effect of relative speed. At slower preferred speeds, the relationship between speed and step width follows a reverse J-curve. As preferred speed increases, the slope flattens out and then becomes positive such that as relative speed increases, step width increases. The consequence of this moderation is that some participants reach a minimum step width at their fastest relative speeds (e.g., preferred = 1.22 m/s), some do not change their step width as relative speed changes (e.g., preferred = 1.27 m/s), and some reach a minimum at their slowest relative speeds (e.g., preferred = 1.72 m/s). The considerable variation across people in this sample indicates that “appropriate” stability moderation differs across people as a function of their preferred speed. Crucially, the mixed effects models used in this design allowed us to tease out the within and between subject variability.

Our results support the notion that the relationship between speed and mediolateral stability is complex^3,15^. Future studies should explore what individual factors contribute to preferred speed and what individual factors affect the relationship between walking speed and mediolateral stability in adults, such as mediolateral pelvis mechanics^16^ or mediolateral and anteroposterior arm swing^17,18^. Future studies may also investigate this relationship in different populations, such as older adults, and with external constraints, such as while dual-tasking or in dim lighting, to determine the effect of stability impairments and task constraints on the variation in the relationship between preferred speed, relative speed and step width.

## Data Availability

The data sets generated during and/or analyzed during the current study are available from the corresponding author on reasonable request.
